# Downregulation of Jumonji-C domain-containing protein 5 inhibits proliferation by silibinin in the oral cancer PDTX model

**DOI:** 10.1371/journal.pone.0236101

**Published:** 2020-07-17

**Authors:** Cheng-Yu Yang, Chang-Huei Tsao, Cheng-Chih Hsieh, Chih-Kung Lin, Chun-Shu Lin, Yu-Hsuan Li, Wei-Chin Chang, Jen-Chen Cheng, Gu-Jiun Lin, Huey-Kang Sytwu, Yin-Lai Wang, Yuan-Wu Chen

**Affiliations:** 1 School of Dentistry, National Defense Medical Center, Taipei, Taiwan, R.O.C; 2 Department of Microbiology and Immunology, National Defense Medical Center, Taipei, Taiwan, R.O.C; 3 Department of Medical Research, Tri-Service General Hospital, National Defense Medical Center, Taipei, Taiwan, R.O.C; 4 Department of Pharmacy Practice, Tri-Service General Hospital, Taipei, Taiwan, R.O.C; 5 Division of Anatomic Pathology, Taipei Tzu Chi Hospital, Taipei, Taiwan, R.O.C; 6 Department of Radiation Oncology, Tri-Service General Hospital, National Defense Medical Centre, Taipei, Taiwan, R.O.C; 7 Graduate Institute of Clinical Medicine, College of Medicine, Taipei Medical University, Taipei, Taiwan, R.O.C; 8 Department of Oral and Maxillofacial Surgery, Tri-Service General Hospital, Taipei, Taiwan, R.O.C; 9 Department of Biology and Anatomy, National Defense Medical Center, Taipei, Taiwan, R.O.C; 10 National Institute of Infectious Diseases and Vaccinology, National Health Research Institutes, Miaoli County, Taiwan, R.O.C; 11 Department of Dentistry, Kaohsiung Armed Forces General Hospital, Kaohsiung, Taiwan, R.O.C; Universidade de Sao Paulo, BRAZIL

## Abstract

Dysregulation of histone demethylase Jumonji-C domain-containing protein 5 (JMJD5) has been identified as a great effect on tumorigenesis. Silibinin is a commonly used anti-hepatotoxic drug and exhibits anticancer effect in various cancers. However, the antitumor mechanism between silibinin and JMJD5 in oral squamous cell carcinoma (OSCC) remains unclear. In this study, the clinical significance of JMJD5 on OSCC patients was assessed through tissue microarray. Furthermore, mice bearing patient-derived tumor xenografts (PDTXs) and tongue cancer cell lines were treated with silibinin and evaluated for tumor growth and JMJD5 expression. High expression of JMJD5 in oral cancer was significantly associated with tumor size (*P* = 0.0241), cervical node metastasis (*P* = 0.0001) and clinical stage (*P* = 0.0002), was associated with worse survival rate compared with that of the total cohort (*P* = 0.0002). Collectively the data indicate that JMJD5 expression may be suitable for detection of unfavorable prognosis in OSCC patients, based in part on its apparent role as a marker of metastasis. In addition, silibinin inhibits cancer growth in vitro and in PDTX models. Furthermore, metastasis-associated protein 1 (MTA1) could regulate the expression for JMJD5 and had a positive correlation with JMJD5. Moreover, silibinin could downregulate JMJD5 and MTA1 in oral cancer. Present study thus identifies that JMJD5 might be an essential prognostic indicator and therapeutic target against OSCC progression. In addition, silibinin is a potential candidate among novel chemotherapeutic agents or adjuvants for modulating JMJD5 in OSCC, through a mechanism likely involving MTA1/JMJD5 axis.

## Introduction

Oral squamous cell carcinoma (OSCC) is a highly aggressive malignant tumor in the world [1, 2]. Despite the therapeutic strategies is improved for OSCC, the 5-year survival rate of early-stage disease is approximately 80% and that of late-stage disease is only 20% [3, 4]. Therefore, novel prognostic factors and treatments are required to improve outcomes of patients with advanced oral cancer [1, 2].

One of the hallmarks of cancer cells is their altered metabolism [5], this generally involves an increased uptake of glucose. In this metabolic flux, the pyruvate kinase muscle isozyme 2 (PKM2) might play an important role [6]. Previously studies revealed that overexpression of PKM2 associates with aggressive clinicopathological features and unfavorable prognosis in OSCC [7, 8]. A study showed that Jumonji-C domain-containing protein 5 (JMJD5, KDM8) regulates nuclear translocation of PKM2 through direct physical binding and hinders the PKM2 tetrameric assembly and causes the nuclear translocation of PKM2 [9].

Aberrations in the local chromatin modification of histone tails cause cancer [10]. Methylated histone lysine residues are a key factor in chromatin remodeling and are demethylated by JmjC-family enzymes [10]. JMJD5 protein is a histone H3K36me2 demethylase and involved in lysine demethylation and hydroxylation functions [9–11]. Studies have been identified that JMJD5 acts in the cyclin A1 coding region to regulate breast cancer cell proliferation [10], and as a dual coactivator of AR and PKM2 integrates AR/EZH2 network and tumor metabolism in prostate cancer [12]. Recently, a study has shown that downregulation of JMJD5 suppresses metastasis and induces apoptosis in OSCC by regulating p53/NF-kappaB pathway [13]. Remarkably, metastasis-associated protein 1 (MTA1) is one of the transcription factors of JMJD5 by using bioinformatics GENECARD web site (www.genecard.com). MTA1 regulates gene expression by functioning as a transcription factor and has been reported associated with cancer progression and poor prognosis [14, 15].

Silybum marianum, commonly known as milk thistle, a member of the asteraceae family, is a therapeutic herb with a 2,000‐year history of use, mainly to treat kidney, spleen, liver, and gallbladder diseases [16–18]. Silibinin is the active component of the medicinal herbal milk thistle [19]. Previously studies have revealed that silibinin have many activities, including in acute and chronic liver injury, growth inhibition, antiproliferative effect, cell cycle arrest, apoptotic induction [20–23], anticancer [24], canceroprotective [25], and inhibition of glycolytic flux [26]. These effects were demonstrated and mediated by its antioxidative, radical-scavenging, others specific receptors interaction, and the modulation of various cell-signaling pathways, including NF-κ B, targeting of STAT3, inhibition of EGFR-MAPK/ERK1/2 signaling, activity upon Rb and E2F proteins, IGF-receptor signaling, etc. [24, 27–29]. Various preclinical studies have shown that silibinin can inhibit lung, breast, gastric, prostate, and skin cancer [20, 21], but this does not reflect in the pilot trials performed in humans to date.

The scarcity of clinically relevant tumor models drastically hinders the development of effective therapies for oral cancer. Establishing PDTX models that truly generalize the phenotypic and genotypic features of oral cancer is critical. PDTX is a creative and innovative preclinical animal model, which consistently illustrates the retained tumor morphology and genetic stability [30, 31]. Briefly, in PDTX models, histologically confirmed OSCC specimens are subcutaneously implanted into NOD.Cg-Prkdcscid Il2rgtm1Wjl/SzJ (NSG) immunodeficiency mice. In vitro and in vivo studies have established that silibinin inhibits several common cancer types, [20, 28, 32]. However, few studies are available on the inhibition effects of silibinin on oral cancer cells [33]. Therefore, this study used a preclinical PDTX model and oral cancer cell line to investigate whether the anticancer effects of silibinin on cell proliferation in oral cancer are exerted through downregulation of MTA1/JMJD5.

## Materials and methods

### Tissue Microarray Construction (TMA)

#### Patients

This study reviewed 87 patients who were diagnosed with malignant OSCC at the Tri-Service General Hospital (Taipei, Taiwan, R.O.C.), from January 2000 to December 2019. This sample included OSCC patients who underwent planned curative primary surgery with or without adjuvant chemoradiotherapy. Exclude other patients with histological diagnosis, including adenocarcinoma, adenoid cystic carcinoma, verrucous carcinoma, adenocarcinoma, sarcoma and mucoepidermoid carcinoma. Patients with metastatic oral cancer, synchronous oral cancer, or other hospital malignancies or treatment history are also excluded. This study was approved by the Institutional Review Committee of Tri-Service General Hospital (TSGH-1-105-05-012 and TSGH-2-105-05-004), and the method was conducted in accordance with the approved guidelines. All subjects obtained informed written consent. The 87 qualified patients include 76 men and 11 women, with ages ranging from 29 to 72 years. According to the 2010 staging criteria of the American Joint Committee on Cancer (AJCC), the pathological stages of all 87 patients were classified [34].The TMA study was described as follow: The hematoxylin and eosin stained slides of all tumors were reviewed by two pathologists. Tumor differentiation of tumors was based on the WHO grading system. The tissue was formalin fixed and paraffin embedded in blocks. The pathologists then marked the areas to be represented in the TMA. One core tissue sample (2 mm) was taken from a representative area of each paraffin-embedded tumor tissue and TMA slides were constructed.

### Cell culture

The SAS (JCRB0260; JCRB), SCC25 (CRL-1628; ATCC), and HSC3 cells are the human oral squamous cell carcinoma cell line. The SAS cell line was provided to us by Dr. Lo (Institute of Oral Biology, Department of Dentistry, National Yang-Ming University, Taipei, Taiwan). The SCC25 cell line was obtained from the American Type Culture Collection (ATCC). The HSC-3 cell line was provided to us by Dr. Yeh (Department of Hematology and Oncology, Cancer Center, Taipei Medical University). All the cell lines were cultured in RPMI 1640 (GIBCO, USA) supplemented with 10% fetal bovine serum (BI, USA), 1% penicillin/streptomycin, and 2 mmol/L L-glutamine in a humidified incubator with a 5% CO_2_ at 37°C.

### Reagents

Silibinin (S0417, Sigma-Aldrich, USA; purity ≥ 98%) was dissolved in dimethyl sulfoxide (DMSO) to form a 100 mM stock and then added to cells at the indicated concentrations. Silibinin and cisplatin (479306, Sigma-Aldrich, USA; purity ≥ 99%) were dissolved in DMSO, polyoxyethylated castor oil (C5135, Kolliphor EL, Sigma-Aldrich, USA), and phosphate-buffered saline (PBS) to be rendered available as a solution for administration in PDTX and xenograft models.

### Plasmids

Specific siRNA targeting MTA1 (stock concentration: 20 μM) was commercially obtained from Dharmacon (Lafayette, CO, USA). For control, a negative control siRNA (scrambled RNA, stock concentration: 20 μM) was used. The plasmids expressing MTA1 were obtained from OriGene (Rockville, MD, USA). Plasmid were transfected using jetPRIME (New York, USA) according to the manufacturer’s instructions, and verified by qPCR assay.

### PDTX establishment and treatment protocol

The protocol for collection of tumor samples from surgically resected T4aN2b stage OSCC to establish PDTXs was approved by the Institutional Review Committee of Tri-Service General Hospital (TSGH-2-105-05-004) and the Institutional Animal Care and Use Committee (IACUC 16–244) of the National Defense Medical Center, Taipei, Taiwan. Patient tumor samples were obtained by informed consent. These methods are in compliance with the ARRIVE guidelines.

PDTX was established using methods described in our previous study [35]. Briefly, Tumor specimens were divided into small pieces (5–10 mm), and implanted into the subcutaneous tissue of the immune deficiency mice (NSG). All mice were bred in in the NDMC animal center. Once tumors reached 1–2 cm in diameter, as measured with calipers, mice were euthanized and tumors were implanted/passaged serially into new NOD SCID mice for at least three times to establish model stability. The tumor volume was calculated as volume = 1/2 × (longest diameters) × (shortest diameters)^2^.

When the tumor volume reached approximately 500 mm^3^, mice with seventh-generation SC179-PDTXs (PDTX for patient No. SC179) were randomized into two groups, one receiving silibinin (200 mg/kg/daily, n = 6) and the other receiving phosphate buffered saline (PBS, vehicle control, n = 5) through intraperitoneal injection for 21 days. Measure body weight and tumor volume at least twice a week.

### OSCC xenograft animal models

The oral cancer SAS xenograft animal model (IACUC 16–244) was used to study the in vivo effects of silibinin on OSCC. Six-week-old non-obese diabetic / severe with immunodeficiency mice (NOD / SCID, NOD.CB17 Prkdc scid / J, National Laboratory Animal Center of Taiwan) were fed at the NDMC Animal Center. Mice were injected with 2 × 10^6^ SAS cells subcutaneously and divided into 3 groups: silibinin (200 mg / kg body weight (BW) / d / intraperitoneal (ip.), n = 6) treatment; positive control (cisplatin, 10 mg / kg BW / d / ip., n = 5); and vehicle control (PBS, n = 5). on the third day, the mice in each group were administered first, and then the treatment continued until the 21st day.

### Histology and immunohistochemistry

The immunohistochemical staining using methods described in our previous study [35, 36]. The following primary antibodies were used: anti-JMJD5 (GTX85251, GeneTex, Taiwan, ROC) and anti-MTA1 (A300-911A, BETHYL, USA) antibodies. The slides were counterstained with hematoxylin (Sigma-Aldrich) and mounted with a mounting solution. An appropriate positive control was included in each series.

### Evaluation of immunohistochemical staining

The intensity of the immunostaining of tumor cells was performed as previously described [35, 36]. Briefly, the protein staining of each slide was scored and evaluated according to the following criteria: 0–3 (0, no staining; 1, weak intensity; 2, medium intensity; 3, strong intensity). In each intensity score, the percentage of tumor cells with nucleus or cytosol staining is graded on a 5-point scale (0, 0%, 1, 0% –25%, 2, 25% –50%, 3, 50%– 75%; and 4, 75% –100%). The immunostaining score (range 0–12) is determined by multiplying the stained tumor cell score (0–4) by the intensity score (0–3). Samples with an immunohistochemistry (IHC) score> 4 were defined as having high JMJD5 expression. In animal studies, the immunostaining score is determined by multiplying the stained tumor cell score (0–4) by the intensity score (0–3) and the percentage of viable tumor cells in the tissue.

### Western blotting

The protein extraction and Western blotting were performed as previously described [35, 36]. Specific antibodies against JMJD5 (GTX85251, GeneTex, Taiwan, ROC), MTA1 (A300-911A, BETHYL, USA), and H3K36me2 (GTX54108, GeneTex, Taiwan, ROC). GAPDH (LF-PA0018, LabFrontier, Korea) was considered as the internal control. The ratio was assessed as the ratio of densities of corresponding protein bands and quantification with Image J software (National Institutes of Health, USA).

### Cell proliferation assay

OSCC cells were plated on a 24-well plate at a density of 20,000 cells/well, grown in culture media containing 10% FBS for overnight and exposed to silibinin (0–250 μM) for 24–48 hrs followed by methylene blue (M9140, Sigma) assay [37]. The absorbance at 590 nm was read on a microplate reader (BioTek).

### Statistical analysis

The collected data were analyzed with GraphPad Prism (GraphPad Software, San Diego, CA, USA) and presented as mean ± standard deviation (SD), and used for all statistical analyses. Unpaired-samples *t* test was performed to compare the differences between tumor and adjacent normal tissue. To examine the various clinicopathological characteristics of JMJD5 expression, the Fisher's exact test was used. Survival analysis was evaluated by Kaplan–Meier plot and log-rank test. The relationship between JMJD5 and MTA1 were measured by correlation analysis. *P* < 0.05 was considered statistically significant.

## Results

### JMJD5 and MTA1 were overexpressed and associated with poor prognosis in oral cancer

**Fig 1:**
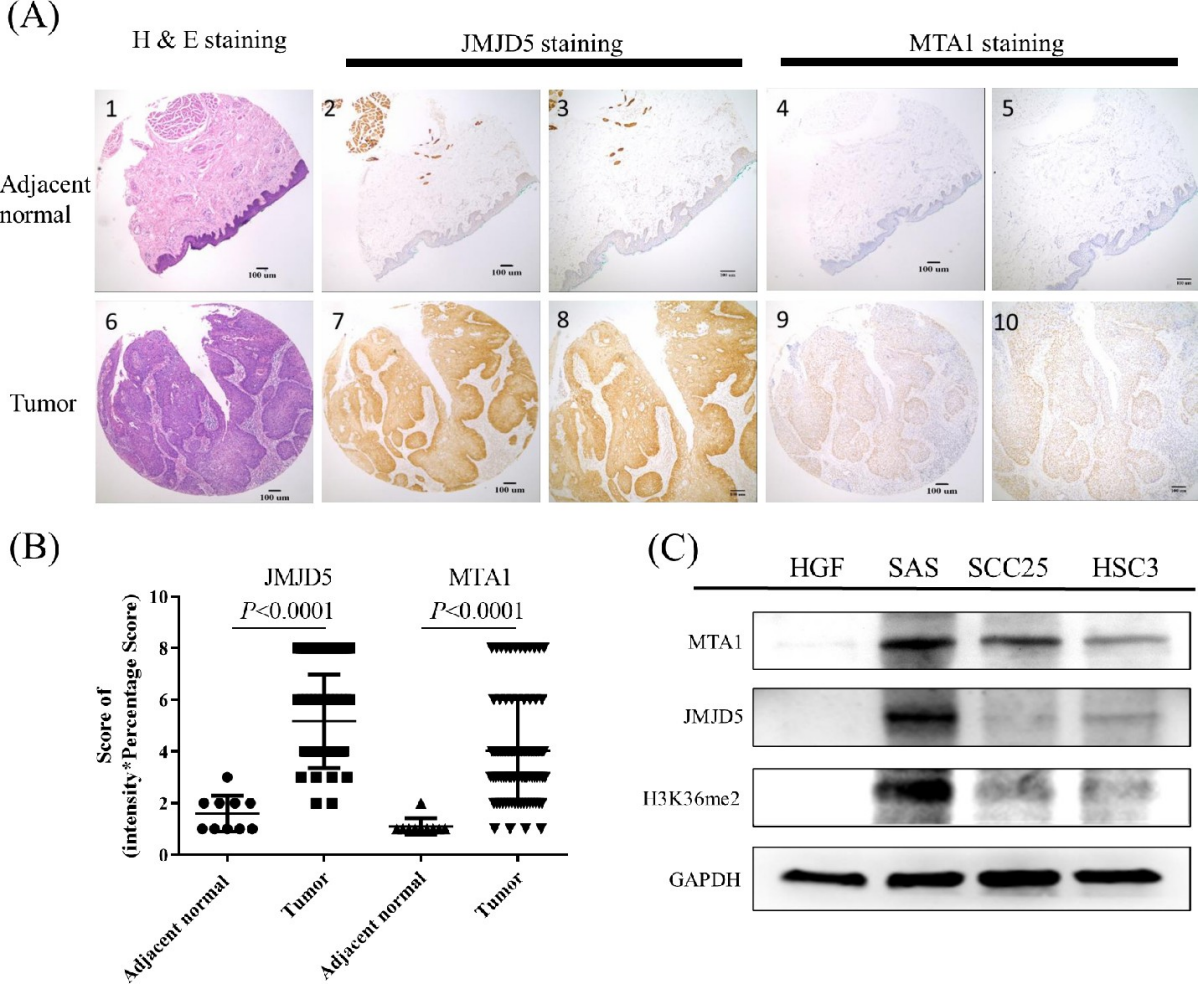
JMJD5 and MTA1 were overexpressed in oral cancer. Representative samples scored for high or low expression for JMJD5 and MTA1 IHC results are shown. (A1) H&E stain for normal tissues adjacent to the tumor (magnification 40x); (A2) JMJD5-stained in normal tissues adjacent to the tumor (magnification 40x); (A3) JMJD5-stained in normal tissues adjacent to the tumor (magnification 100x); (A4) MTA1-stained in normal tissues adjacent to the tumor (magnification 40x); (A5) MTA1-stained in normal tissues adjacent to the tumor (magnification 100x); (A6) H&E stain for OSCC tumor tissue (magnification 40x); (A7) JMJD5-stained in OSCC tumor tissue (magnification 40x); (A8) JMJD5-stained in OSCC tumor tissue (magnification 100x); (A9) MTA1-stained in OSCC tumor tissue (magnification 40x); (A10) MTA1-stained in OSCC tumor tissue (magnification 100x). (B) JMJD5 and MTA1 were highly expressed in oral cancer tissues. The score was defined intensity score x percentage score. Data are expressed as mean ± standard deviation. *P* < 0.05 (Student’s t-test) is represented significance difference. (C) Both JMJD5 and MTA1 were high expressed in the oral cancer cell lines than in normal cells. HGF as a primary cultured normal human gingival fibroblast cell. H3K36me2, the di-methylation at the 36th lysine residue of the histone H3 protein.

To determine the role of JMJD5 in oral cancer, the protein levels of JMJD5 and MTA1 in 87 oral cancer and adjacent normal tissues were measured with IHC staining. The tumor tissues showed higher JMJD5 and MTA1 expression levels compared with adjacent normal tissues (*P* < 0.001, Fig 1A and 1B and Table 1). Furthermore, OSCC cell lines revealed higher JMJD5 and MTA1 expression levels compared with normal human gingival fibroblast (Fig 1C).

**Table 1:** Associated between JMJD5 expression and multiple clinicopathological parameters in OSCC.

Clinicopathological parameters		Cases	JMJD5		*p*-values
			Low	High	
**Normal oral mucosa**		10	10	0	**0.0002**
**OSCC**		87	34	53	
**Gender**					
	Male	76	28	48	0.3273
	Female	11	6	5	
**Age**					
	≦52	53	20	33	0.8233
	>52	34	14	20	
**Tumor size**					
	T1-T2	53	26	27	**0.0241**
	T3-T4	34	8	26	
**Cervical node metastasis**					
	N(-)	41	25	16	**0.0001**
	N(+)	46	9	37	
**Clinical stage**					
	I-II	32	21	11	**0.0002**
	III-IV	55	13	42	
**Recurrent**					
	R(-)	43	21	22	0.0808
	R(+)	44	13	31	

Bold font indicates significance (*P* < 0.05).

Clinicopathological data of 87 patients with oral cancer used in the study.

**Fig 2:**
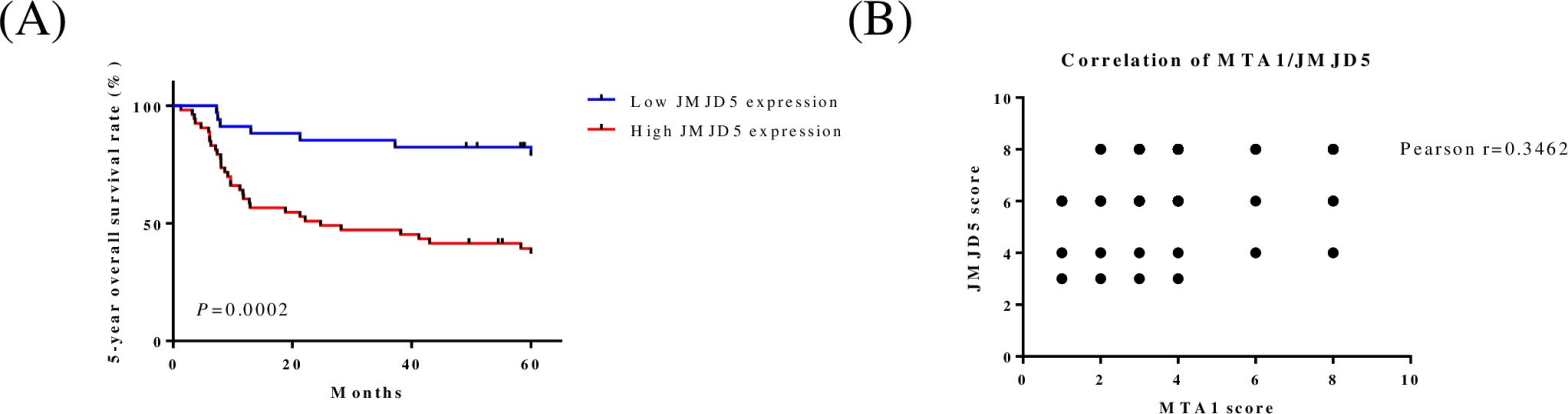
High JMJD5 expression was associated with poor prognosis for oral cancer patients. (A) The Kaplan–Meier curve compares the 5-year overall survival of oral cancer patients with high- or low-level JMJD5 protein products. Samples that have IHC scores >4 are defined as having high JMJD5 expression. (B) Correlation analysis of JMJD5 and MTA1 expression in OSCC tissue microarray.

To evaluate JMJD5 status in oral cancers, we assessed the expression of JMJD5 by OSCC tissue microarrays (n = 87; Fig 1A and 1B and Table 1) containing different grades of oral cancer and adjacent normal tissues, and the stained positive cells was calculated as described previously [35]. The expression of JMJD5 in tumor tissues was significantly higher than in normal oral mucosa (*P* = 0.0002; Table 1). In addition, the high expression levels of JMJD5 were associated with tumor size (*P* = 0.0241; Table 1), cervical node metastasis (*P* = 0.0001; Table 1), clinical stage (*P* = 0.0002; Table 1), and five-year overall survival rate (*P* = 0.0002; Fig 2A). Furthermore, we observed a positive correlation between JMJD5 and MTA1 (r = 0.3462; Fig 2B).

### Overexpression of MTA1 induced JMJD5 expression and cell proliferation in oral cancer

**Fig 3:**
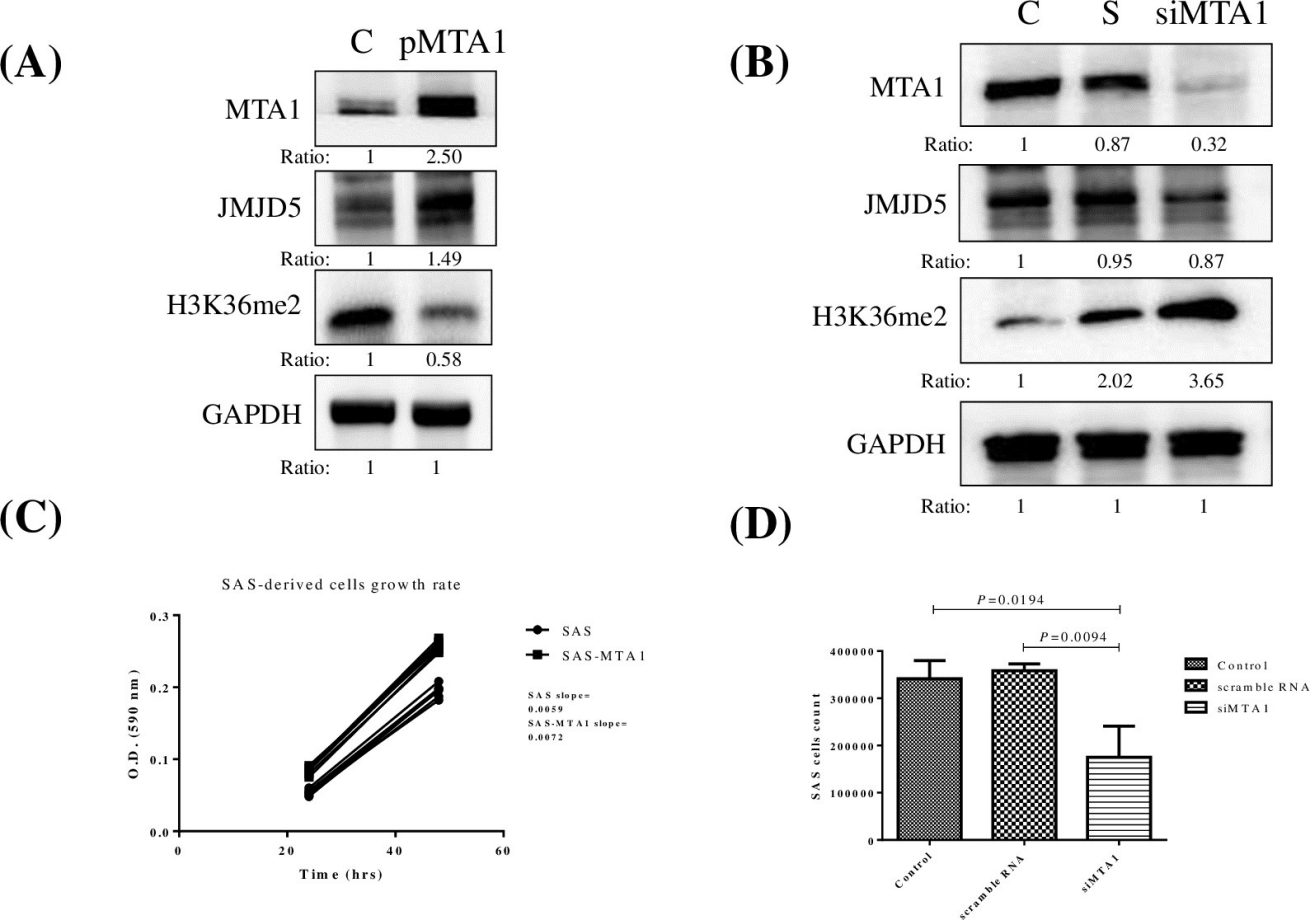
MTA1 overexpression induced JMJD5 expression and cell proliferation in oral cancer. (A) Constructs expressing MTA1 were developed and transfected into SAS cells. C as the parental SAS cells without transfected MTA1 expressed vector. pMTA1 as transfected overexpressed MTA1 vector. Overexpressed MTA1 upregulated JMJD5 and downregulated H3K36me2. (B) MTA1 knockdown downregulated JMJD5 and upregulated H3K36me2. The ratio was assessed as the ratio of densities of corresponding protein bands and quantification with Image J software. (C) Overexpressed MTA1 promoted the growth of oral cancer SAS cells. (D) Repression of MTA1 using siRNA suppressed SAS cell proliferation. S as the scramble RNA. The concentration of S and siMTA1 are 20 μM.

MTA1 is one of the transcription factors of JMJD5 by bioinformatics tools analysis on GENECARD website. To verify the relation between JMJD5 and MTA1 in oral cancer, cells expressing MTA1 constructs were developed and transfected into SAS cells. Overexpressed MTA1 upregulated JMJD5 and downregulated H3K36me2 (Fig 3A). Moreover, MTA1 knockdown downregulated JMJD5 and upregulated H3K36me2 (Fig 3B). In accordance with slope value for cell proliferation assay, overexpressed MTA1 promoted the growth rate of oral cancer SAS cells (Fig 3C). Conversely, when MTA1 expression was inhibited with siRNA, SAS cell proliferation was suppressed (Fig 3D).

### Silibinin inhibited oral cancer growth and downregulated JMJD5 and MTA1

**Fig 4:**
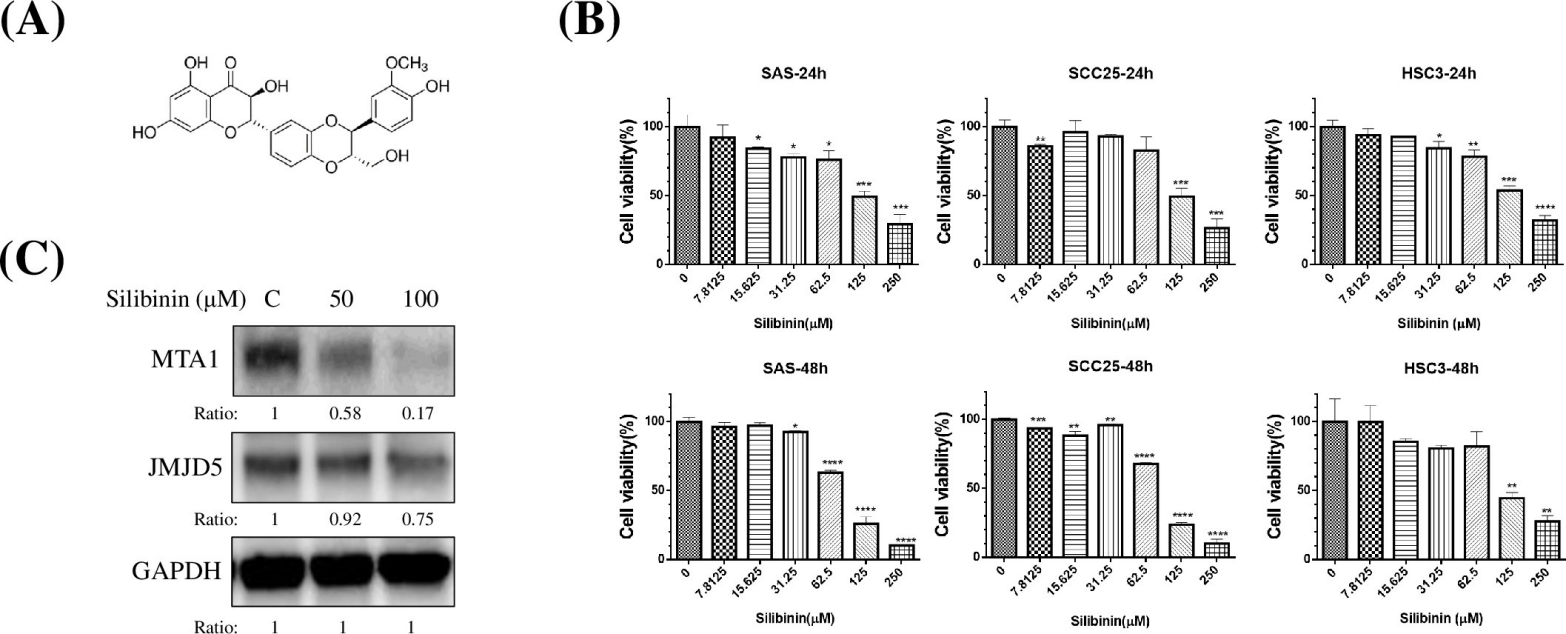
Silibinin inhibited the oral cancer growth and downregulated both JMJD5 and MTA1. (A) Structure of silibinin. (B) Silibinin inhibited the oral cancer cell growth in a time- and dose-dependent manner (*P* < 0.05). (C) Silibinin inhibited the expression of both JMJD5 and MTA1 in SAS cells at 48hrs. The ratio was assessed as the ratio of densities of corresponding protein bands and quantification with Image J software.

Silibinin is an herb-active compound (Fig 4) and has an anticancer effect in various cancers, but its efficacy is unknown in case of oral cancer. We found that silibinin inhibits the oral cancer cell growth in a time- and dose-dependent manner (Fig 4B, *P* < 0.05). The IC_50_ was 100 μM approximately for SAS cells at 48hrs. In this study, silibinin downregulated the expression of both JMJD5 and MTA1 in SAS cells at 48hrs (Fig 4C).

### Silibinin inhibited oral cancer growth in patient-derived tumor xenograft

**Fig 5:**
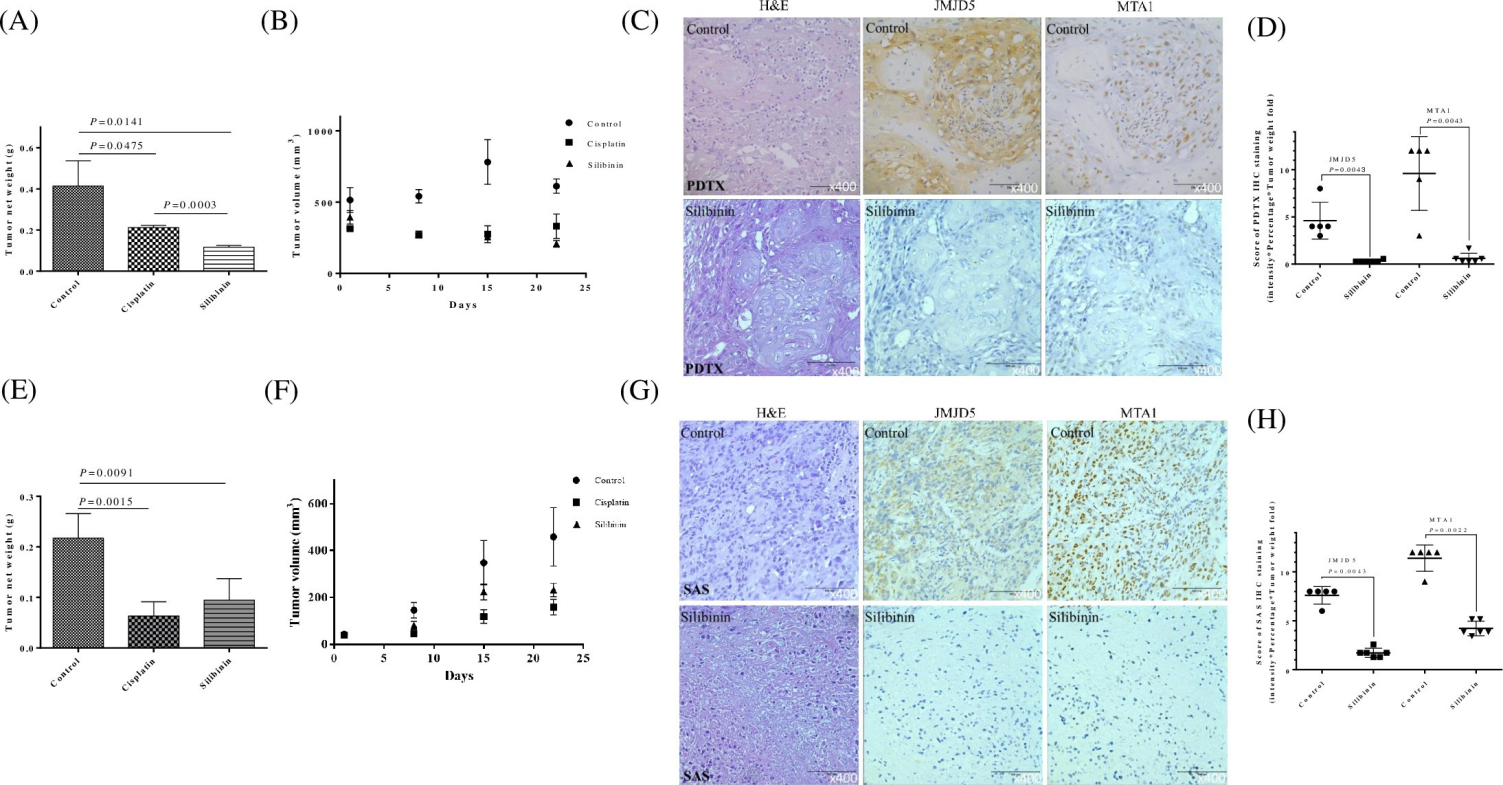
Silibinin inhibited oral cancer growth and downregulated both JMJD5 and MTA1 in vivo and in PDTX models. (A) Changes in tumor weight in SC179 oral cancer PDTX models treated for 21 days with silibinin (200 mg/kg daily i.p., n = 6), cisplatin (10 mg/kg, n = 5), and PBS (a vehicle control, n = 5). (B) Changes in tumor volume in SC179 oral cancer PDTX models treated for 21 days with silibinin (200 mg/kg daily i.p.), cisplatin (10 mg/kg), and PBS (a vehicle control). Tumor volumes were compared with those of controls. All data are expressed as mean ± SD. *P < 0.05 (Student *t* test). (C) H&E staining and IHC were performed after the administration of silibinin or PBS (a vehicle control). In total, SC179 oral cancer PDTX models stained positive for JMJD5 and MTA1. Immunodetectable proteins are stained brown; nuclei are counterstained blue. Original magnification, 400×. (D) Immunostaining scores of JMJD5 and MTA1. The intensity of tumor cell immunoreactivity was scored on a scale of 0–3: 0, no staining; 1, weak intensity; 2, moderate intensity; and 3, strong intensity. The percentage of tumor cells with nucleus staining for each intensity score was graded on a 5-point scale: 0, 0%; 1, 0%–25%; 2, 25%–50%; 3, 50%–75%; and 4, 75%–100% stained tumor cells. The immunostaining scores (range 0–12) were determined by multiplying stained tumor cells scores (0–4) with intensity scores (0–3). (E) SAS cells were subcutaneously xenografted into NOD/SCID mice. Changes in the tumor weight of SAS xenograft models treated for 21 days with silibinin (200 mg/kg daily i.p., n = 6), cisplatin (10 mg/kg, n = 5), and PBS (a vehicle control, n = 5). (F) Changes in tumor volume in SAS xenograft models treated for 21 days with silibinin (200 mg/kg daily i.p.), cisplatin (10 mg/kg), and PBS (a vehicle control). Tumor volumes were compared with those of controls. All data are expressed as mean ± SD. *P < 0.05 (Student *t* test). (G) H&E staining and IHC were performed after administration of silibinin or PBS (a vehicle control). SAS xenograft models stained positive for JMJD5 and MTA1. Immunodetectable proteins are stained brown; nuclei are counterstained blue. Original magnification, 400×. (H) The immunostaining scores of JMJD5 and MTA1. The intensity of tumor cell immunoreactivity was scored on a scale of 0–3: 0, no staining; 1, weak intensity; 2, moderate intensity; and 3, strong intensity. The percentage of tumor cells with nucleus staining for each intensity score was graded on a 5-point scale: 0, 0%; 1, 0%–25%; 2, 25%–50%; 3, 50%–75%; and 4, 75%–100% stained tumor cells. The immunostaining scores (range 0–12) were determined by multiplying scores based on the percentages of stained tumor cells (0–4) with the intensity scores (0–3). *P* < 0.05 (Student *t* test) means statistically significant.

We examined the anticancer effects of silibinin in preclinical oral cancer PDTX models. We found that silibinin inhibited tumor growth compared with the vehicle control (PBS) in the SC179-PDTX model (Fig 5A and 5B), where cisplatin was the positive control. No apparent toxicity or weight loss was observed in the mice after silibinin administration during the experimental period.

Abnormal activation of JMJD5 and MTA1 contributes to tumor survival, growth, and metastasis. IHC analysis showed that JMJD5 and MTA1 are expressed in oral cancer SC179-PDTX, and these expressions were significantly downregulated after silibinin administration in clinical tumor-bearing mice compared with controls (Fig 5C and 5D).

The anti-cancer effects of the silibinin were further investigated in SAS tumor xenografts model. After SAS cells were transplanted into NOD/SCID mice, the daily treatments were initiated. The doses for individual treatments (for 21d) are outlined as follows: silibinin, 200 mg/kg/d/i.p.; cisplatin (first-line drug for oral cancer treatment), 10 mg/kg/d/i.p. The silibinin and cisplatin significantly repressed tumor growth in NOD/SCID mice (Fig 5E and 5F), and the bodies of the mice were not changed.

The expression levels of JMJD5 and MTA1 were analyzed through IHC staining (Fig 5G and 5H). Statistical analysis showed that the expression levels of JMJD5 and MTA1 were significantly decreased in the silibinin-treated group than in the untreated control groups.

## Discussion

Drug repurposing or off-label use of medicines is widespread in several diseases [38]. Such a prescribing practice has been particularly more acute in oncology [39]. However, as the clinical benefits and potential toxicities of anticancer drugs are uncertain, off-label use of anticancer drugs remains a limitation.

Patient-derived xenograft models generated from human tumor samples that retain donor tumor histology and genetic characteristics can be used as personalized medicine mouse models; compared to other traditional preclinical models, such models are used in translational cancer research It is more desirable [40] and can be used to accelerate the development of targeted anti-tumor drugs and the discovery of biomarkers [31, 41].

Milk thistle has a long history of being used to treat liver and biliary complaints; however, it was not until 1968 that silibinin (silymarin) was isolated from the seeds of the plant, and it was proposed that silibinin might be the active ingredient [42]. Although several studies have reported that silibinin exerts good anticancer effect through multiple pathways [25, 32], no human clinical trial has been conducted using milk thistle or silibinin as cancer treatment or adjunctive therapy. Silibinin has been reported to inhibit oral cancer cell proliferation [43] and migration [33] in vitro and in vivo.

In this study, we developed the JMJD5-overexpressing PDTX model by using cancer cells of a patient with advanced OSCC, and we showed that silibinin downregulates JMJD5 and inhibits cell growth in vitro and in vivo (Figs 4 and 5) and in the PDTX model (Fig 5) of oral cancer. According to our results, the anticancer activity of silibinin is as good as that of cisplatin in PDTX (Fig 5) and in vivo (Fig 5). Thus, silibinin is a valuable anticancer drug for OSCC treatment.

Despite the advanced therapeutic strategies for OSCC, the 5-year survival rate of patients is approximately 50% [44]. Therefore, novel prognostic factors and treatments must be established to prevent oral cancer progression. Studies have shown that JMJD5 is an oncogene in lung, breast, colon, and prostate cancer [45–48], but the role of JMJD5 in oral cancer is not defined. A recent study revealed that JMJD5 was upregulated in patients with oral cancer, and JMJD5 downregulation suppressed metastasis and induced apoptosis through regulation of the p53/NF-κB pathway [13]. In this study, we found that JMJD5 was overexpressed in patients with oral cancer and in OSCC cell lines (Fig 1), and it was associated with tumor size, cervical lymph nodes metastasis, clinical stage, and 5-year overall survival rate (Fig 2B), further supporting the role of JMJD5 as a potential prognostic factor in OSCC progression.

JMJD5 knockdown inhibited the proliferation of colon, breast, and oral cancer cells [13, 47, 48]. By using bioinformatics tools (GENECards, https://www.genecards.org), we found that MTA1 is one of the transcription factors for JMJD5. Studies have revealed that MTA1 played an oncogenetic role in cancers, including head and neck cancer [14, 15, 49]. In this study, MTA1 upregulation caused JMJD5 induction and OSCC cell proliferation (Fig 3A and 3C). Conversely, MTA1 knockdown caused JMJD5 inhibition and proliferation in OSCC cells (Fig 3B and 3D). Furthermore, according to IHC assay, JMJD5 was correlated with MTA1 in patients with oral cancer (Fig 2C). Therefore, MTA1 inhibition could suppress JMJD5 expression in OSCC cells, thus preventing OSCC progression.

Various plant extracts, including tea polyphenol, resveratrol, ginger extract, and soy isoflavones, have demonstrated potential antitumor effects, which may provide novel strategies for investigating potential anticancer drugs. Silibinin is an herbaceous botanical extract and exhibits potent antioxidant, immunomodulatory, antifibrotic, antiproliferative, and antiviral activities, although the mechanism of action is incompletely understood [20, 50, 51]. Studies have revealed that JMJD5 downregulation inhibits cancer growth [10, 45, 52]. In addition, MTA1 repression inhibited cancer cell growth [14, 15]. Furthermore, studies have shown that JMJD5 is associated with cancer metabolism [9, 10, 53]. Notably, silibinin has been reported to interfere with cancer metabolism [54]. In this study, we found that silibinin inhibited oral cancer growth in vitro, in vivo, and in preclinical oral cancer PDTX models (Figs 4 and 5). We also found that silibinin could downregulate both JMJD5 and MTA1 (Fig 4C).

In conclusion, JMJD5 was frequently highly expressed and associated with poor prognosis in OSCC. JMJD5 suppression markedly reduced cancer cell proliferation at least partly through the regulation of MTA1 signals. In addition, silibinin suppressed oral cancer cell proliferation through downregulation of JMJD5 and MTA1 in vitro, in vivo, and in PDTX. However, the signaling pathway through which silibinin represses JMJD5 and MTA1 in oral cancer cells remains unknown. Furthermore, silibinin is a potential chemotherapeutic agent for oral cancer. Finally, this study lays a foundation for further evaluation of the action mechanism and preclinical trial of silibinin against oral cancer.

## Supporting information

S1 Raw images (PDF)

S1 Checklist (PDF)

S1 Table Multivariate analyses of prognostic factors related to 5-year overall survival OSCC in the JMJD5-stained high score group. (DOCX)
