# Supplementary material for: Downregulation of Jumonji-C domain-containing protein 5 inhibits proliferation by silibinin in the oral cancer PDTX model
Source: PLoS One. 2020 Jul 17;15(7):e0236101. doi: 10.1371/journal.pone.0236101 (PMC7367477; doi:10.1371/journal.pone.0236101)
Supplement: S1 Raw images — (PDF) [file pone.0236101.s001.pdf]

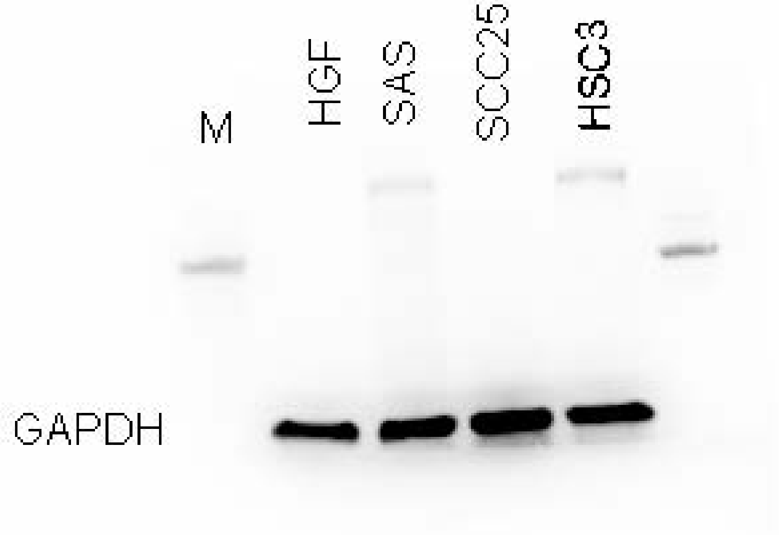

Fig1C

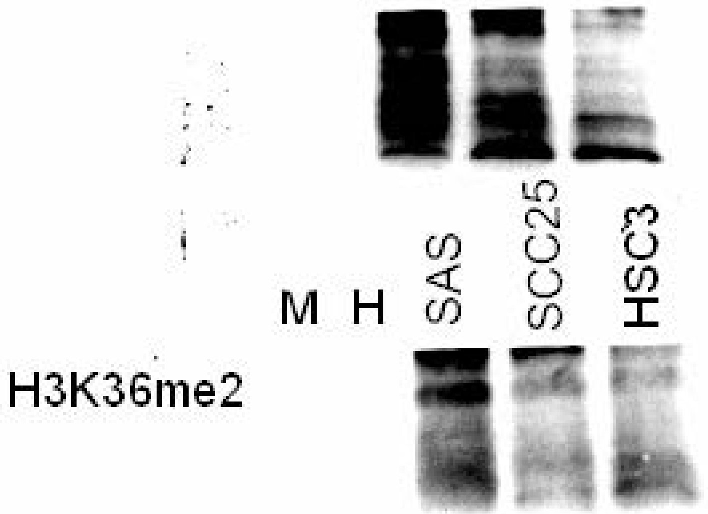

Fig1C

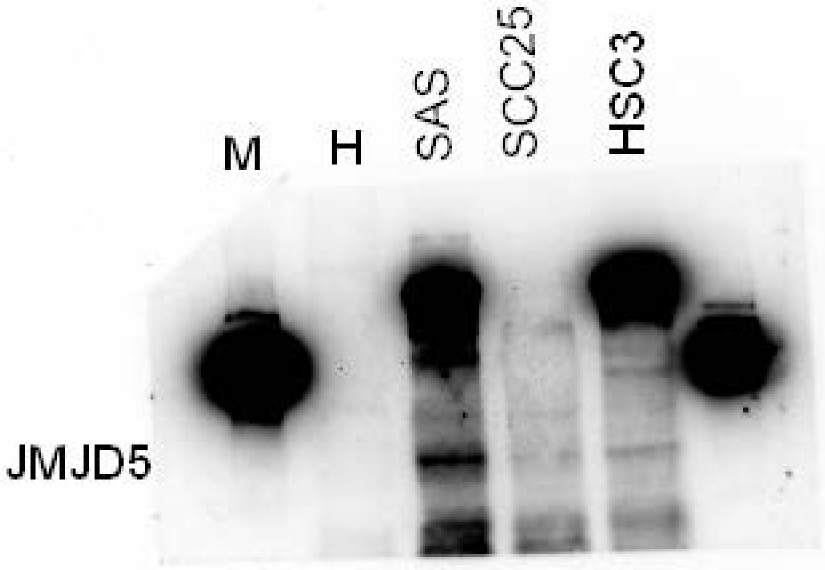

Fig1C

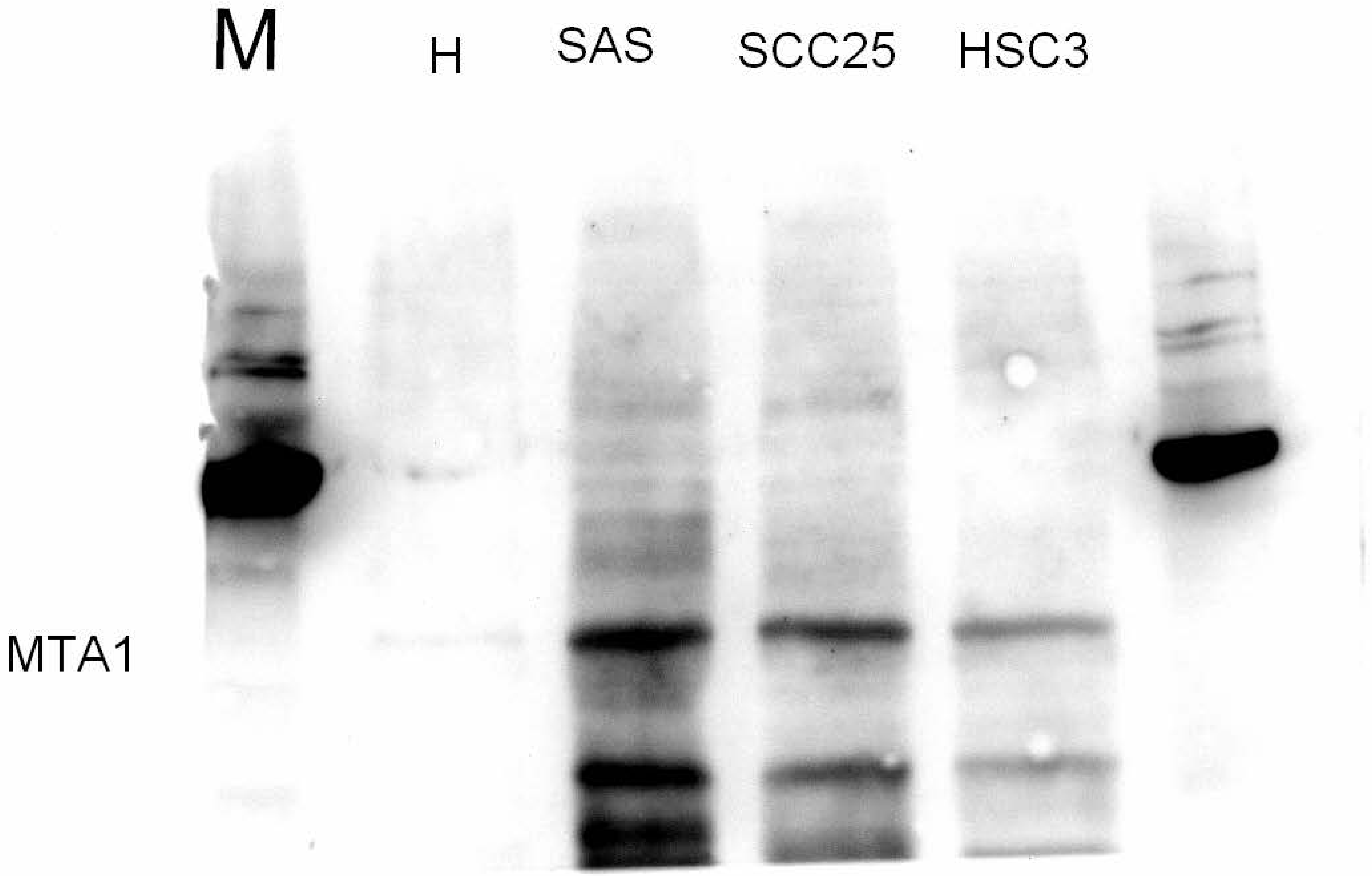

Fig1C

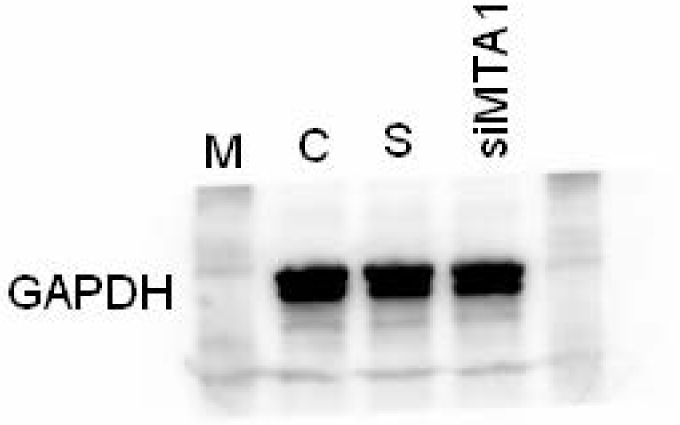

Fig3B

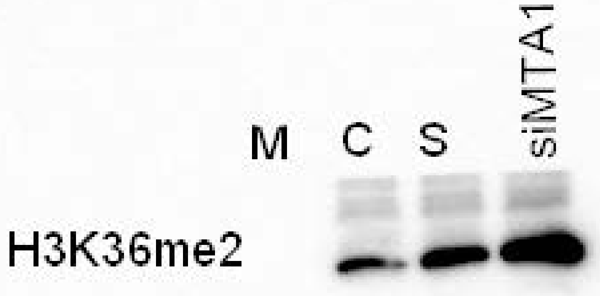

Fig3B

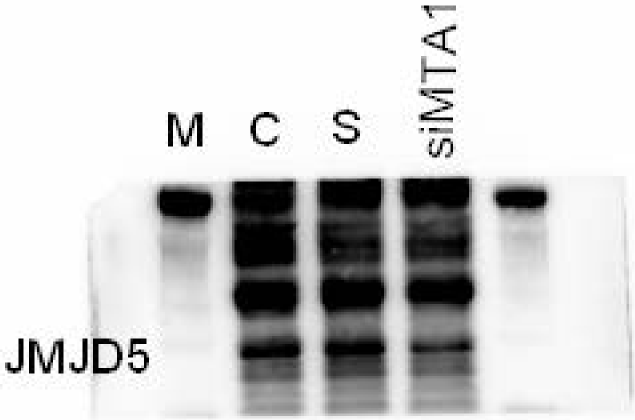

Fig3B

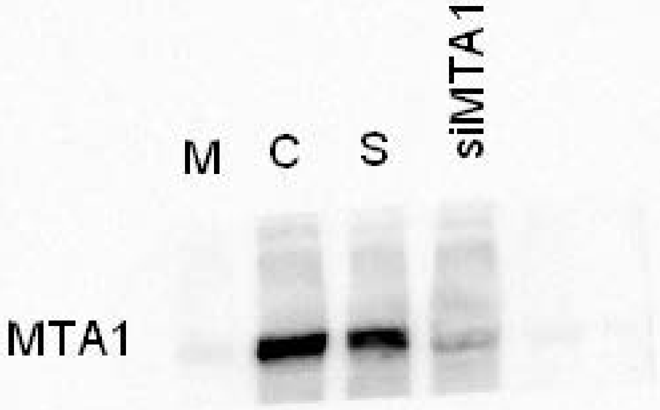

Fig3B

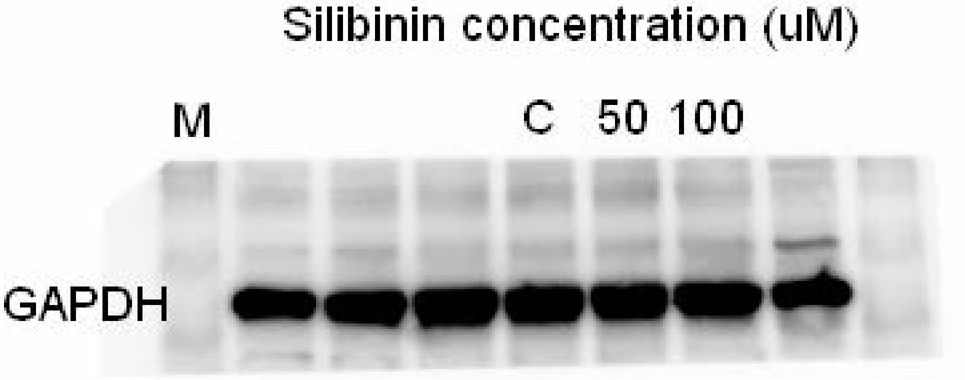

Fig4C

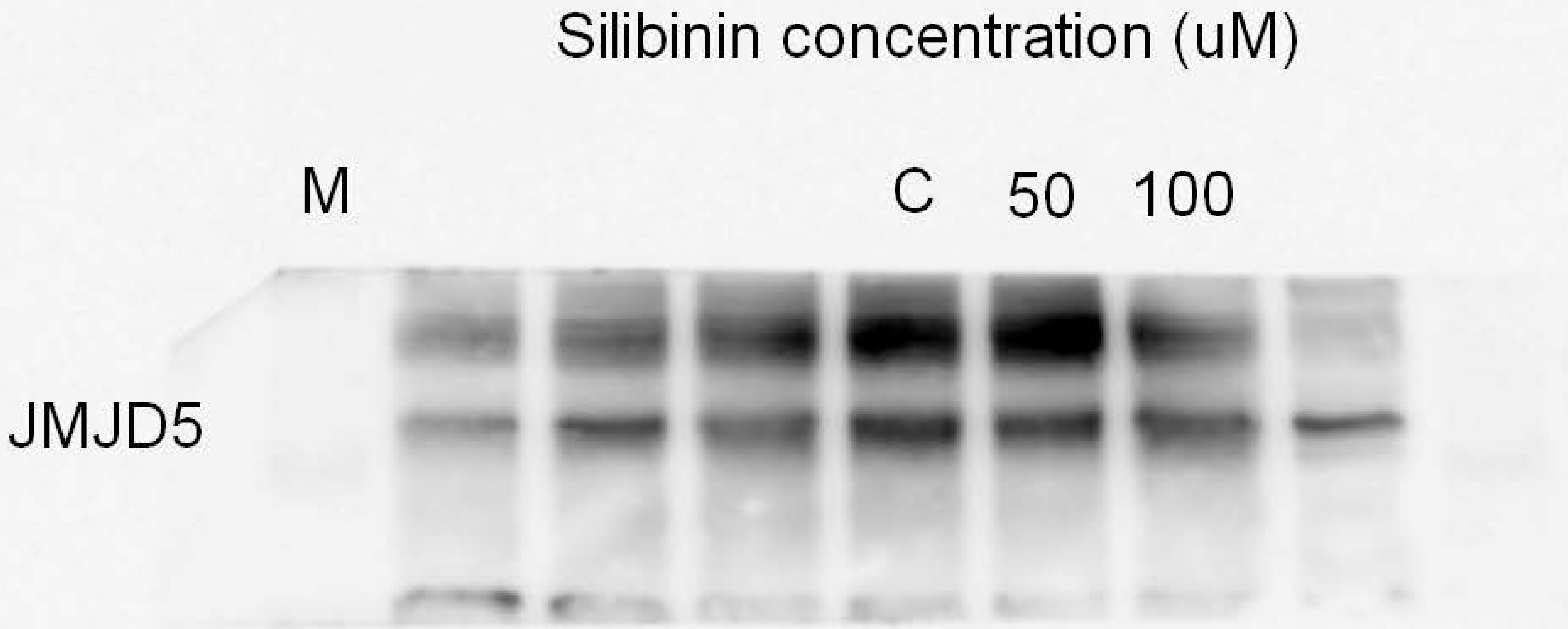

Figure 4C

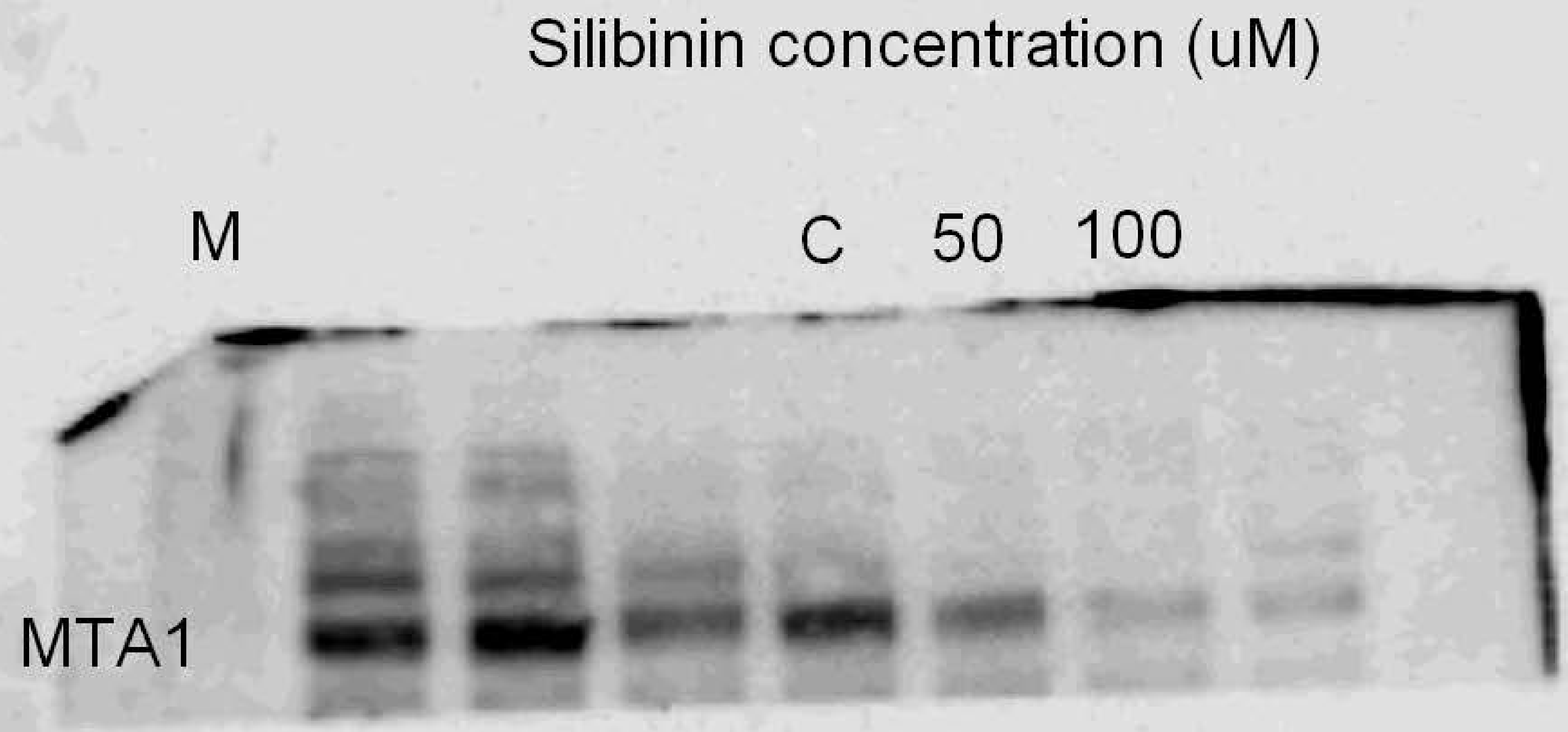

Figure 4C

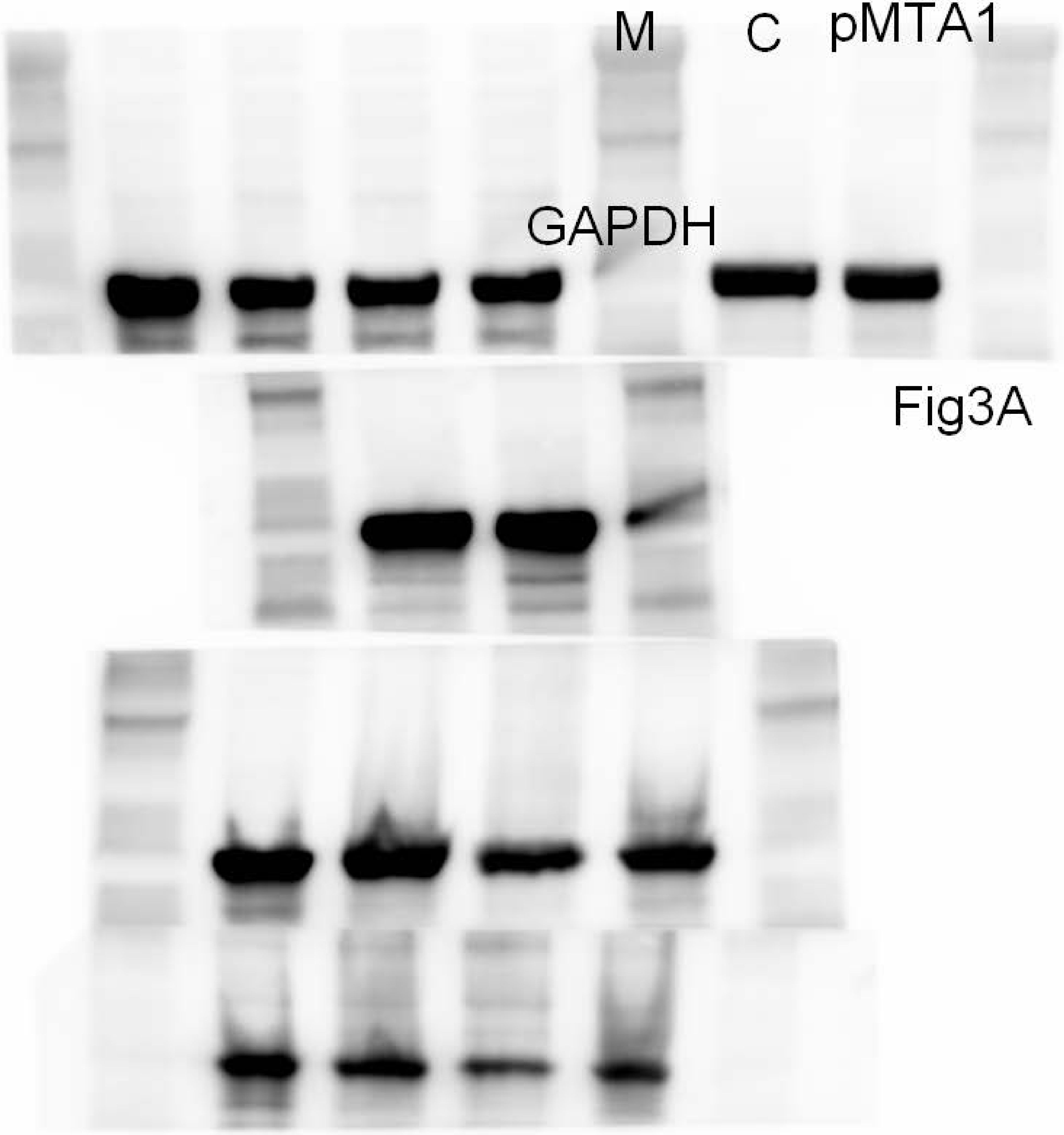
M
C
pMTA1
GAPDH
Fig3A

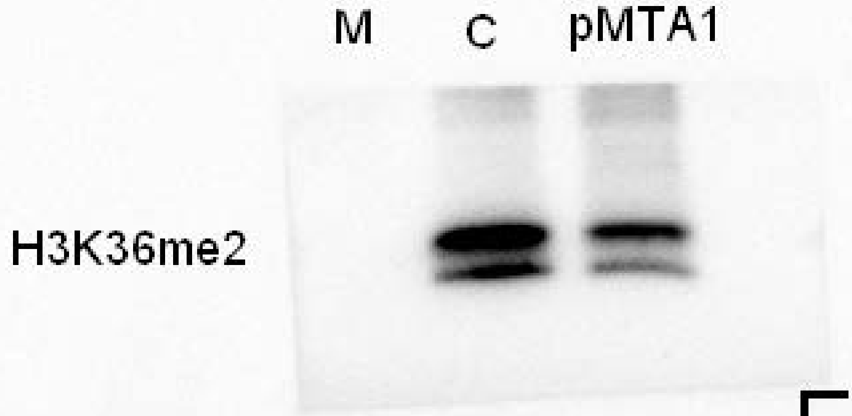

Fig3A

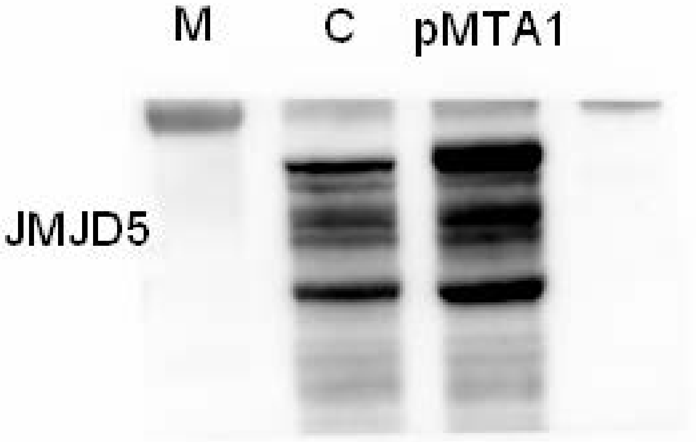

Fig3A

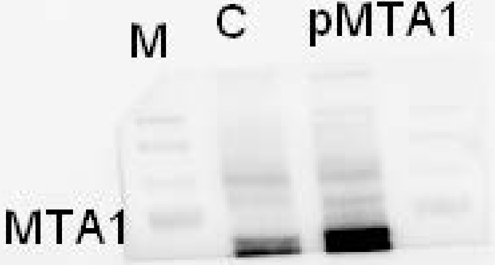

Fig3A
