# Supplementary material for: Downregulation of Jumonji-C domain-containing protein 5 inhibits proliferation by silibinin in the oral cancer PDTX model
Source: PLoS One. 2020 Jul 17;15(7):e0236101. doi: 10.1371/journal.pone.0236101 (PMC7367477; doi:10.1371/journal.pone.0236101)
Supplement: S1 Table — (DOCX) [file pone.0236101.s003.docx]

**Table S1.** Multivariate analyses of prognostic factors related to 5-year overall survival OSCC in the JMJD5-stained High score group.

|  | Multivariate Analysis | |
| --- | --- | --- |
| **Characteristics**  **(n, %)** | HR  (95% CI) | *P* |
| Sex |  | 0.930 |
| Female (11, 12.6) | Reference |  |
| Male (76, 87.4) | 1.058(0.299-3.740) |  |
| Age, years |  | 0.783 |
| ≤52 (53, 60.9) | Reference |  |
| >52 (34, 39.1) | 1.102(0.554-2.189) |  |
| JMJD5-stained Score |  | **0.022*** |
| ≤4 (Low) (34, 39.1) | Reference |  |
| >4 (High) (53, 60.9) | 2.957(1.167-7.493) |  |
| Nod metastasis |  | 0.158 |
| N (-) (41, 47.1) | Reference |  |
| N (+) (46, 52.9) | 2.150(0.743-6.225) |  |
| Tumor Size |  | **0.044*** |
| T1 (18, 20.7) | Reference |  |
| T2 (35, 40.2) | 1.879(0.211-16.699) | 0.571 |
| T3 (7, 8.1) | 5.549(0.594-51.817) | 0.133 |
| T4 (27, 31) | 10.726(1.071-107.434) | **0.044*** |
| Clinical stage |  | 0.261 |
| S1 (13, 14.9) | Reference |  |
| S2 (19, 21.8) | 0.720(0.048-10.756) | 0.811 |
| S3 (17, 19.5) | 0.866(0.062-12.176) | 0.915 |
| S4 (38, 43.7) | 0.253(0.015-4.186) | 0.337 |

* Indicates a significant difference, *p* <0.05.

CI, confidence interval; HR, hazard ratio.
